# Paradigm shift in apomixis research: Targeting mutants with elements of apomixis in sexual crops

**DOI:** 10.1007/s00425-026-05030-x

**Published:** 2026-05-30

**Authors:** Samela Draga, Giovanni Gabelli, Fabio Palumbo, Lucia Colombo, Emidio Albertini, Fulvio Pupilli, Gianni Barcaccia

**Affiliations:** 1 Department of Agronomy, Food, Natural resources, Animals and Environment, University of Padova, 35020 Legnaro, PD Italy; 2 Department of Biosciences, University of Milan, 20133 Milan, Italy; 3 Department of Agricultural, Food and Environmental Sciences, University of Perugia, 06121 Perugia, Italy; 4 Institute of Biosciences and Bioresources, National Research Council (CNR), 06128 Perugia, Italy

**Keywords:** Apomixis, Paradigm shift, Natural mutants, Crops, Agricultural resilience

## Abstract

**Main conclusion:**

In addition to model system studies and synthetic engineering, prioritizing the discovery of naturally occurring apomixis-like mutants in crops would provide an immediate route to applied innovation in plant breeding.

**Abstract:**

Apomixis is a remarkable form of asexual reproduction that enables plants to produce seeds without meiotic reduction or gametic fertilization in ovules. This process is mainly divided into two types: sporophytic and gametophytic apomixis, each following a distinct embryogenetic pathway with specific processes and characteristics of significant importance in plant biology. Apomixis provides insight into plant reproductive strategies, facilitating genetic stability and adaptation without reliance on sexual reproduction. An in-depth understanding of these pathways not only enriches knowledge in plant biology but also holds potential implications for advancing agricultural practices and conservation efforts. Yet current research on apomixis follows two main directions: (1) identifying and characterizing genes in natural model systems, and (2) engineering synthetic apomixis by introducing genes that mimic key components of asexual reproduction. Here, we sustain the screening and investigation of naturally occurring mutants that may arise in populations from spontaneous DNA changes (*i*.*e*., mutants that are not induced artificially through genetic engineering or by molecular or cellular biotechnological methods), with possible elements of apomixis directly in crop plant species. Such a perspective may lead to new opportunities to harness apomixis for crop improvement and the development of resilient varieties, as well as to accelerate the transfer and preservation of desirable plant traits.

## Introduction—aspects of apomixis

**Fig. 1:**
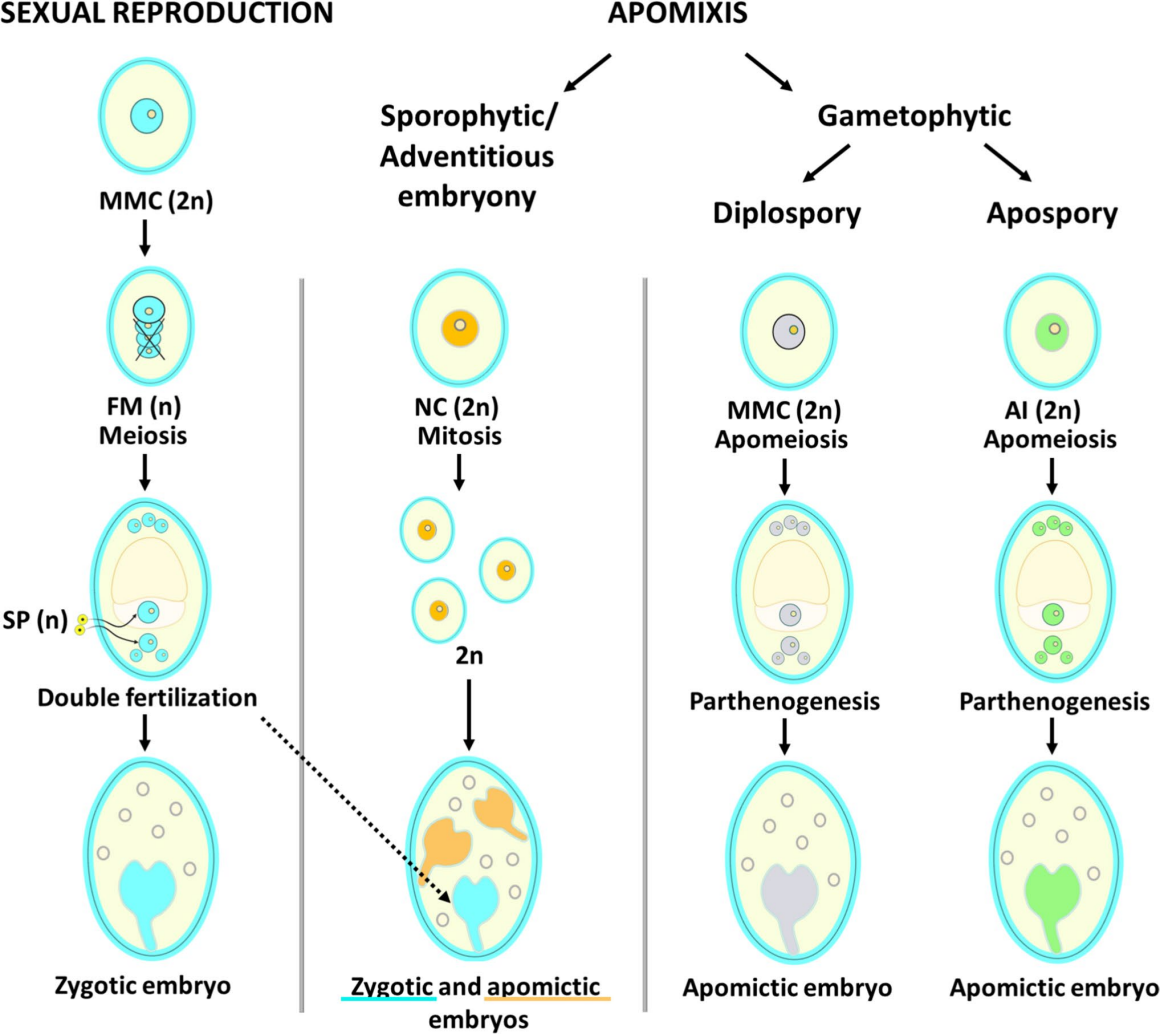
Main elements of the embryogenetic pathway of amphimixis (sexual reproduction) and apomixis (asexual reproduction), including sporophytic (adventitious embryony, often resulting in sexual and apomictic embryos in one seed; polyembryony) and gametophytic (diplospory and apospory) types. *MMC*, megaspore mother cell; *FM*, functional megaspore; *SP*, sperm cell; *NC*, nucellar cell (sporophytic origin); *AI*, aposporous initial cell (nucellar cell)

Apomixis refers to a type of asexual reproduction that allows plants to avoid meiotic reduction and fertilization, resulting in the production of clonal seeds and progenies that are genetically identical to the mother plant (Koltunow et al. 1995a; Spillane et al. 2001; Ortiz and Pessino 2002; Richards 2003). This process is categorized primarily into two distinct types: sporophytic and gametophytic apomixis. Each type exhibits a unique pathway, characterized by specific processes, similarities, and differences that underscore their significance in plant biology (Fig. 1).

The direct development of an embryo from sporophytic cells, typically in the nucellar region of the ovule, without forming an embryo sac, is named sporophytic apomixis (Richards 2003; Naumova 2018; Hörandl 2024). The initiating event is the formation of a multinucleate cell, which bypasses the gametophytic stage entirely. These sporophytic cells can undergo mitotic divisions to produce a globular embryo that subsequently differentiates into a mature seed. Apomictic embryos often develop alongside zygotic (sexually derived) embryos (see Fig. 1), indicating that apomixis and amphimixis may occur simultaneously (Ozias-Akins 2006). However, during seed development, adventitious embryos at the micropylar end often develop most rapidly and dominate the zygotic embryo, resulting in progeny genetically identical to the mother plant (Spillane et al. 2001).

In contrast, the initial stage of gametophytic apomixis involves megasporogenesis. Notably, the megaspore mother cell (diplospory) or one of the nucellar cells (apospory) develops into an unreduced and clonal embryo sac. In diplosporic apomixis, the megaspore mother cell forms megaspores via incomplete meiosis or mitosis (Fig. 1) (Cornaro et al. 2025). Aposporic apomixis is based on the formation of a clonal embryo sac by mitosis, starting from the nucellus, *i*.*e*., the aposporous initial cell (see Fig. 1) (Mazzucato et al. 1995; Galla et al. 2011; Wang and Underwood 2023; Hörandl 2024). After the formation of the embryo sac, the egg cell develops into an embryo by parthenogenesis without fertilization (Koltunow 1993, 1994; Burson 1994; Koltunow and Grossniklaus 2003). Endosperm can develop autonomously or by fertilization of the central cell of the embryo sac (pseudogamous apomixis); in either case, it has an unbalanced ratio of parental genomes, different from the canonical 2 (maternal): 1 (paternal) that allows sexual seeds (especially cereals) to be viable (Rojek and Ohad 2023; Terzaroli et al. 2023). Importantly, the apomictic embryo is genetically identical to the maternal plant, thereby preserving the genetic lineage of the parent. As a consequence, gametophytic apomixis is characterized by the direct formation of an embryo from gametophytic tissues, typically resulting in offspring that mirror the genetic makeup of the parent.

The differences between the two forms of apomixis are notable and primarily revolve around the sources from which embryos are formed: sporophytic apomixis presents a more straightforward approach by bypassing the gametophyte stage altogether, whereas gametophytic apomixis typically involves a more intricate developmental pathway, leading to the formation of a functional gametophyte (see Fig. 1) (Koltunow and Grossniklaus 2003; Hojsgaard and Hörandl 2015; Xu et al. 2021).

Despite their distinct pathways, sporophytic and gametophytic apomixis share fundamental similarities. Both processes are forms of asexual reproduction in plants that enable seed production without recombination and fertilization, resulting in offspring that are clones of the maternal plant (Pessino et al. 1999; Grimanelli et al. 2001; Koltunow and Grossniklaus 2003). This inherent capacity facilitates genetic uniformity, as both pathways produce seeds containing genetic material identical to that of the maternal plant. Additionally, both forms lead to the production of viable seeds and progeny that possess the potential to fix and inherit parental plant traits (Spillane et al. 2001; Richards 2003; Ozias-Akins and Conner 2020; Wang and Underwood 2023).

In any case, apomictic reproduction is never completely independent from sexuality; i) natural apomictic plants are rarely obligate, as some sexuality persists and its penetrance depends mainly on environmental conditions; and ii) male gametes are generally reduced and recombinant, although exceptions have been reported, such as in *Boechera* (Mau et al. 2013, 2021). Such residual sexuality constitutes a reservoir of genetic variability in natural apomictic populations otherwise committed to an evolutionary dead end due to the possible accumulation of deleterious mutations (van Dijk and Vijverberg 2005; Hörandl and Paun 2007; Hojsgaard and Hörandl 2015; Sailer et al. 2020). Plasticity in sexual/asexual reproduction in angiosperms can accelerate breeding programs in which superior genotypes produced in segregating populations can be readily fixed by apomixis (Ozias-Akins 2006; Barcaccia and Albertini 2013; Jank et al. 2025). However, asexual reproductive pathways analogous to apomixis are not restricted to angiosperms, as in ferns and other pteridophytes, apogamy, where the sporophyte develops directly from somatic cells of the gametophyte without fertilization, is present (Chao et al. 2012; Liu et al. 2012; Grusz 2016; Marimuthu et al. 2022). From an evolutionary perspective, numerous studies have been employed in this front, highlighting significant developmental plasticity of reproductive programs, which may reflect evolutionarily conserved mechanisms, multiple independent origins, and/or potential links to abiotic stress as a trigger for the establishment of asexual reproduction (Beck et al. 2012; Dyer et al. 2012; Haufler et al. 2016; Grusz et al. 2021; Hornych et al. 2021; Picard et al. 2021).

To conclude, apomixis offers valuable insights into plant reproductive strategies, enabling genetic stability and adaptation without sexual reproduction. An in-depth understanding of these pathways not only enriches the knowledge in plant biology, but also has potential implications for advances in agricultural practices and conservation efforts (Schmidt 2020, 2025; Jank et al. 2025; Karunarathne et al. 2025). By elucidating these mechanisms, researchers can better explore how apomictic processes can be harnessed in crop species. In fact, apomixis presents significant opportunities for breeding and maintaining plants with favorable traits, given its capacity to stabilize any elite genotype (Ozias-Akins 2006; Fiaz et al. 2021). Nevertheless, our understanding of the genetic origins and control of naturally occurring apomictic systems in plants remains poorly understood, and strategies based on individual gene manipulation have shown limited success (Hojsgaard 2020; Ren and Sundaresan 2026).

### Apomixis research last 50 years: achievements and limitations

**Fig. 2:**
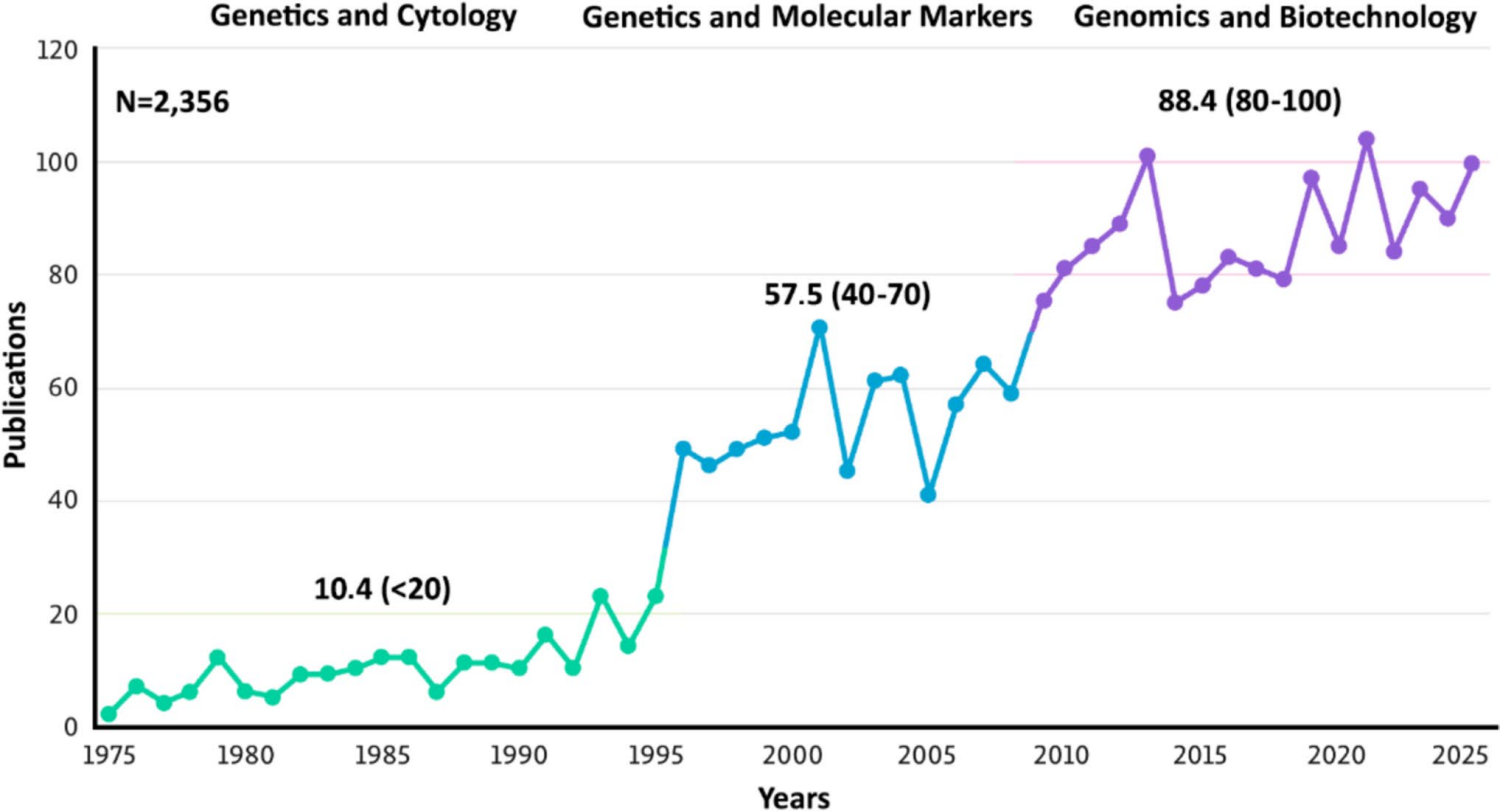
Trends in apomixis research publications over the past 50 years (1975–2025), categorized by the predominant technologies applied. The early phase (1975–1995) was dominated by genetics and cytology approaches (< 20 publications/year, mean = 10.4). A second phase (1995–2010) coincided with the development of molecular markers, with an increase to ~ 40–70 publications/year (mean = 57.5). Since 2010, the genomics and biotechnology era has driven a further rise, averaging ~ 80–100 publications/year (mean = 88.4). The dataset includes a total of 2,356 publications. Scopus database, accessed on 05 July 2025

Over the past five decades, research on apomixis has steadily grown, with notable advances in technological methods (Fig. 2). Current research on apomixis mainly focuses on two areas: (1) identifying and characterizing genes that control apomixis in natural model systems, and (2) engineering synthetic apomixis by introducing genes that mimic key steps of asexual reproduction into crop species. The first area has led to studies of apomixis-related candidate genes in model species, such as *Paspalum*, *Poa pratensis*, *Eragrostis curvula****,**** Cenchrus, Pilosella* (*Pilosella piloselloides*, previously *Hieracium pilosella*), *Hieracium*, *Taraxacum*, and *Boechera* (Koltunow et al. 1998; van Dijk and Bakx-Schotman 2004; Albertini et al. 2005; Okada et al. 2007; Sharbel et al. 2009; Vijverberg et al. 2010; Corral et al. 2013; Kotani et al. 2014; Siena et al. 2014, 2016, 2022; Bräuning et al. 2018; Mancini et al. 2018; Colono et al. 2019, 2022; Zühl et al. 2019; Marconi et al. 2020; Carballo et al. 2021b; Bakin et al. 2022; Pasten et al. 2022; Cornaro et al. 2023, 2025), and, to a lesser extent, to exploring apomictic potential in non-model species, such as sunflower, castor bean, walnut, strawberry guava, and alfalfa (Barcaccia et al. 2000a; Palumbo et al. 2024; Setayeshnasab et al. 2024, 2025; Bao et al. 2025; Da Luz-Graña et al. 2025; Lv et al. 2025; Pessino et al. 2025). Despite the discovery of apomixis-linked loci in various species, only a few apomixis-related genes have been functionally studied in natural apomicts, including *APOLLO* in *Boechera*, (Honari et al. 2024), *TRIMETHYL GUANOSINE SYNTHASE 1-TGS1* and *QUI-GON JINN (QGJ)* in*Paspalum*, (Colono et al. 2019; Mancini et al. 2018) for apospory/diplospory; *ASGR-BBML* in *Cenchrus*, (Conner et al. 2017), *PARTHENOGENESIS (PAR)* in *Taraxacum*, (Underwood et al. 2022) for parthenogenesis; and *ORIGIN OF REPLICATION COMPLEX* Sub.3 (*ORC3*) in *Paspalum*, (Bellucci et al. 2023) for unbalanced endosperm development.

Conversely, research on *Arabidopsis* mutants (*fis*, *mea*, *fie*) has provided insights into the mechanisms, showing that autonomous endosperm can result from deregulation of the *Polycomb* repressive gene complex in the central cell (Luo et al. 2000; Spillane et al. 2000). In addition, *TGS1* has recently been demonstrated in *Arabidopsis* to be a candidate gene associated with apomeiosis (Siena et al. 2023; Goeckeritz et al. 2024). However, no definitive genes controlling apomeiosis or autonomous endosperm formation have been confirmed so far. This remains a key challenge, emphasizing the need to identify and validate the functions of natural genes involved in apomixis.

On a different front, advances in synthetic apomixis and efforts to incorporate elements from natural apomicts into sexually reproducing crops have led to a significant increase in scientific interest, as evidenced by the growing number of publications focused on biotechnological approaches (see Fig. 2, post-2010). Over the past five years, research output on synthetic apomixis has followed a clear trend: nearly half of the publications (24 of 47; Scopus accessed: 15 October 2025) are review papers (including crop-oriented ones, such as rice and wheat). The prominence of review papers highlights rising enthusiasm for applying apomixis to crop improvement, while also indicating that this field remains largely conceptual or exploratory rather than marked by significant practical achievements.

So far, genetically modified plants expressing synthetic apomixis have been developed by incorporating genes that control meiosis and parthenogenesis (Underwood et al. 2022; Vernet et al. 2022; Cornaro et al. 2023; Li et al. 2023; Wei et al. 2023; Xiong et al. 2023; Huang et al. 2024; Liang and Gao 2024; Song et al. 2024; Hu et al. 2025). The main focus has been on research involving genes, such as *Mitosis instead of Meiosis* (*MiMe*), a genetic tool to induce apomeiosis, and *Baby Boom 1* (*BBM1*) or *PAR*, transcription factors expressed from the male (sperm) allele immediately after fertilization and essential for initiating embryo development (Conner et al. 2015, 2017; Mieulet et al. 2016; Khanday et al. 2020; Chen et al. 2022; Underwood et al. 2022).

Synthetic apomixis, despite being an innovative breakthrough in plant biotechnology aimed at combining apomeiosis, parthenogenesis, and autonomous endosperm development, remains largely unexplored in major crops (Wang 2020; Fiaz et al. 2021; Underwood et al. 2022; Vernet et al. 2022; Li et al. 2023; Mahlandt et al. 2023; Wang and Underwood 2023; Wei et al. 2023). Although most investigations have focused on rice and reaching significant results, substantial challenges persist (Khanday et al. 2019; Wang et al. 2019; Zhao et al. 2021; Purwestri et al. 2023; Dan et al. 2025; Hu et al. 2025). For recent advances in synthetic apomixis, see the latest reviews published over the past five years (Zhang et al. 2020; Fiaz et al. 2021; Khanday and Sundaresan 2021; Underwood and Mercier 2022; Xu et al. 2022; Li et al. 2023; Mahlandt et al. 2023; Xiong et al. 2023; Goeckeritz et al. 2024; Qu et al. 2024; Sivasankarreddy et al. 2024; Awan et al. 2025; Heidemann et al. 2025; Ji et al. 2025; Reed et al. 2025; Schmidt 2025). Challenges in fully reconstructing the apomictic pathway remain, as genetic engineering efforts often induce apomeiosis and/or parthenogenesis, but fail to achieve coordinated autonomous endosperm formation, leading to early embryo arrest (Vijverberg et al. 2019; Fiaz et al. 2021). The trait is also regulated by complex genetic and epigenetic networks, and each crop likely requires a distinct engineering approach; for example, attempts to engineer *MiMe* in cotton resulted in a loss of fertility (Qian et al. 2024). These outcomes underscore how difficult and time-consuming it can be to transfer strategies effective in model systems, such as *Arabidopsis,* or rice to crops with larger or polyploid genomes (Heidemann et al. 2025). The recent review by Heidemann et al. (2025) provides a thorough discussion of key bottlenecks and intriguing open questions. Overall, these challenges highlight that although synthetic apomixis holds great promise, its practical application in agriculture is still in the early stages.

### Toward a new paradigm in apomixis research: screening for apomixis elements in crops

After five decades of research on apomixis, we believe that it is time to diversify the efforts of the scientific community by exploring new opportunities beyond the induction of synthetic apomixis and the study of genes governing apomixis in wild aposporic or diplosporic models (e.g., *Hieracium*, *Paspalum*, *Urochloa*, *Taraxacum*, *Hypericum*, *Boechera*, *Cenchrus*, *Megathyrsus, Eragrostis*, *Erigeron*, *Pilosella*, *Poa*, *Ranunculus*, *Dryopteris*). In other words, we argue that apomixis research has now reached a stage where apart from studying model systems and synthetic approaches, it is equally important to explore elements of apomixis directly within crop species, as a complementary direction.

In this review, we aim, as an initial step, to emphasize the importance of thoroughly examining the existing scientific literature and the geobotanical context, alongside theoretical evolutionary frameworks, when assessing natural elements of apomixis in sexually reproducing crops. Here, we focus exclusively on natural elements of apomixis and spontaneously occurring mutations, excluding outcomes derived from genetically transformed or biotechnologically engineered mutants. Future efforts toward the practical implementation of this third approach would involve large-scale screening strategies, flow cytometric seed screening (FCSS) of extensive germplasm collections, cytological analysis of ovule development, progeny tests to assess clonality and reproductive mode, and systematic mining of germplasm repositories.

Identifying apomictic elements in major crops could simplify registration processes and make it easier to navigate the complex regulatory system for genetically modified organisms (GMOs). The regulatory framework for approving new plant varieties in the European Union, especially those created through genome editing (*i.e.*, synthetic apomicts), remains highly complicated and contentious (Andersen 2025; Dolezel et al. 2025; Mundorf et al. 2025). Differing opinions among EU institutions, ongoing scientific debates, patent disputes, and societal concerns have significantly slowed the approval process for genome-edited crops (Boora et al. 2025). In this context, the regulatory implications of shifting apomixis research toward large-scale screening within crop populations are significant. If new varieties could be developed through traditional breeding by utilizing apomictic processes, they would fall outside the scope of restrictive EU regulations (organisms derived from New Genomic Techniques, specifically NGT-2 type). Although these concepts remain speculative, a balanced strategy that leverages conventional plant breeding by exploiting large-scale collections of plant genetic resources may offer a more feasible and socially acceptable approach to incorporating apomixis into crop varieties. This strategy could ultimately be supported by NGTs, including genome editing methods. 

Furthermore, compared to natural apomicts, crop plants benefit from extensive genomic and transcriptomic resources, which could accelerate the identification of apomixis-related genes and mechanisms if apomictic lines in crops could be identified. High-quality genome assemblies, pangenome datasets, transcriptome atlases, and mutant collections already exist for several staple crops and could assist in identifying candidate genes, regulatory networks, and epigenetic mechanisms associated with apomixis.

Importantly, focusing on crops could also have broader implications for the research community. By expanding beyond specialized model systems, apomixis research could bring together expertise and new perspectives while increasing funding opportunities, as crop-focused projects often align more closely with agricultural policy priorities and global food security agendas. In this way, crop-focused apomixis research could foster a more inclusive and applied scientific community, accelerating both potential discoveries and significant outcomes.

Finally, we propose a perspective that does not aim to replace or diminish the importance of studies in traditional model apomictic systems, which have laid the foundation for the field. Instead, we suggest a complementary, but different approach involving crop-focused screening efforts that can be pursued alongside model-based studies and synthetic approaches. We believe that through the integration of these strategies and the collaborative expertise of the apomixis research community, the potential of apomixis advancements in agriculture may be achieved.

As a first step to illustrate this concept, we summarize in the following sections apomictic elements in crop species, categorized into sporophytic and gametophytic types. In this review, we refer to ‘crop’ as plant species that are intentionally cultivated for agrifood, pharmaceutical, and animal feed purposes, based on their direct economic value and potential breeding efforts to improve them. Additionally, we highlight the availability of genomic and transcriptomic data in these crop species compared to the main model species used so far for apomixis research.

### Sporophytic apomixis in crops: natural apomicts and mutants with elements of apomixis

Sporophytic apomixis, as mentioned in the introduction, is a form of asexual seed formation in which embryos develop from sporophytic cells of the ovule. This process often results in polyembryony, with one sexually derived zygotic embryo developing alongside multiple clonal embryos genetically identical to the maternal plant (Koltunow 1993; Koltunow et al. 1995a; Richards 2003; Bicknell and Koltunow 2004; Ozias-Akins 2006; Bicknell and Catanach 2015). Notably, sporophytic apomixis is particularly common in tropical tree species that produce high-value fruits, where it appears to have evolved as a reproductive ‘backup’ mechanism, as Richards (2003) suggests. Most studies reporting advances in sporophytic apomixis have been conducted in *Citrus* species. Outside *Citrus*, sporophytic apomixis has been reported in several tropical and subtropical species (Richards 2003; Hojsgaard et al. 2014). The main reports for natural occurrence of sporophytic apomixis and mutants with elements of sporophytic apomixis are listed below and summarized in Table 1.

**Table 1:** List of crops with natural occurrence of sporophytic apomixis and mutants with elements of sporophytic apomixis

Species name	Plant family	Features	Ploidy	Polyembryony Frequency	Methodologies	References
Agrifood						
Grapefruit, orange, lemon (*Citrus* spp.)*	Rutaceae	Eudicot, Tree, Hermaphrodite flowers, Perennial	Diploid	Depending on genotype, 50–90%	Molecular markers, histological analysis, flow cytometry, transcriptomic analysis, progeny test	(Koltunow et al. 1995b; Garcia et al. 1999; Aleza et al. 2010; Kishore et al. 2012; Wang et al. 2017, 2022; Jia et al. 2021, 2023; Martínez-Ochoa et al. 2021; Wu et al. 2021; Tan et al. 2025)
Magenta cherry (*Syzygium paniculatum*)	Myrtaceae	Eudicot, Tree, Hermaphrodite flowers, Perennial	Diploid, Tetraploid	NR	Seed dissection, embryo counting, molecular markers	(Thurlby et al. 2011, 2012)
Mango (*Mangifera indica*)*	Anacardiaceae	Eudicot, Tree, Hermaphrodite flowers, Perennial	Diploid	Up to 97%	Seed dissection and embryo counting, molecular markers	(Litz and Schaffer 1987; Aron et al. 1998; Ochoa et al. 2012; Yadav et al. 2023; Ali et al. 2025)
Mangosteen (*Garcinia mangostana*) Kokum (*Garcinia indica*)	Clusiaceae	Eudicot, Tree, Polygamodioecious, Perennial	Diploid	36–87%	Prevention from pollination in gynoecious plants, molecular markers, segregation analyses	(Horn 1940; Dike et al. 2020; Baskaware and Deodhar 2023)
Papaya (*Carica papaya*)	Caricaceae	Tree-like herb, Dioecious and hermaphrodite, Perennial	Diploid	61% of the flowers set fruits	Prevention from pollination in gynoecious plants, histological analysis	(Vegas et al. 2003; Acevedo-Benavides and Bolaños-Villegas 2021)
Pepper (*Capsicum annuum*)	Solanacee	Eudicot, Herbaceous, HermaphrodIte, Annual	Diploid	NR	Progeny tests	(Nowaczyk 1987)
Prickly pear (*Opuntia ficus-indica*)	Cactaceae	Eudicot, Succulent shrub, Perfect flowers, Perennial	Polyploid	0.5–20%	Molecular markers, progeny test	(Mondragon-Jacobo 2001; Felker et al. 2010; Kaaniche-Elloumi et al. 2013)
Sichuan pepper (*Zanthoxylum bungeanum, Z. armatum*)	Rutaceae	Eudicot, Tree, Dioecious, Perennial	Polyploid	NR	Histological, transcriptomic analyses	(Fei et al. 2021b, a; Wang et al. 2021; Ang 2025)
*Pharmaceutical*						
Guggul (*Commiphora wightii*)	Burseraceae	Eudicot, shrub-small tree, Dioecious, Perennial	Polyploid	1.1–50%	Prevention from pollination, histological analysis, flow cytometry	(Gupta et al. 1996, 1998; Geetha et al. 2013; Kavane et al. 2021, 2022)

*Natural apomicts

*NR* not reported

### Agrifood

#### *Citrus* spp.

Among cultivated species, *Citrus* spp. (including sweet orange, lemon, and grapefruit) represent one of the best-studied examples of sporophytic apomixis (Garcia et al. 1999; Xu et al. 2021; Wang et al. 2022; Wang and Underwood 2023). Many genotypes exhibit high levels of nucellar embryony, with typically 50–90% of seeds containing clonal embryos; however, apomictic ability highly depends on the genotype, and some cultivars are fully sexual (Aleza et al. 2010). Early histological and developmental studies outlined the origin and timing of nucellar embryo formation (Koltunow et al. 1995b), while molecular mapping approaches identified genetic loci associated with apomixis in *Citrus* × *Poncirus* hybrids (Garcia et al. 1999). Recent transcriptomic and regulatory analyses have shed light on the gene expression patterns underlying nucellar embryogenesis, revealing similarities to somatic embryogenesis pathways (Long et al. 2016; Xu et al. 2021). Due to its economic value, abundant genomic resources, and genetic variability in apomictic expression, *Citrus* has become a model for studying the mechanisms of sporophytic apomixis. From a genetic perspective, a locus responsible for citrus polyembryony has been mapped to an 80-kb region containing 11 candidate genes. Among them, the most promising is *CitRWP*, which is expressed at higher levels in the ovules of polyembryonic cultivars. A Miniature Inverted-repeat Transposable Element (MITE) insertion in the promoter region of *CitRWP* was found to co-segregate with polyembryony (Wang et al. 2017). Ongoing research has shown that DNA hypermethylation may activate *CitRWP*, leading to increased *C2H2* expression and ROS levels. This is thought to trigger epigenetic regulation, guiding cell fate changes and establishing the identity of nucellar embryo initial cells in apomictic tissues (Jia et al. 2021, 2023). A recent study by Tan et al. (2025) supported the hypothesis of a close relationship between polyembryonic development and oxidative stress, since overexpression of *CitRWP* led to significant enrichment of genes involved in ROS metabolism, redox regulation, and antioxidant defence.

#### Magenta cherry (*Syzygium paniculatum*)

*S. paniculatum*, a rare Australian rainforest tree, is known to exhibit polyembryony, with embryos arising from maternal nucellar tissue (Lughadha and Proença 1996; Thurlby et al. 2011, 2012). Indeed, genotyping studies suggest that the species is polyploid and exhibits extremely low genetic diversity (Thurlby et al. 2012). In polyembryonic seeds with more than two embryos (one of which is sexual), sexual seedlings do not exhibit superior fitness compared to the apomictic ones. Typically, the largest embryo (apomictic) within a seed germinates first, gaining a competitive advantage over co-occurring embryos (Thurlby et al. 2012).

#### Mango (*Mangifera indica*)

In mango, the frequency of polyembryony can reach 97%, and this trait appears to be controlled by a single dominant gene (Aron et al. 1998). A recent genome-wide association study identified a locus on chromosome 17 linked to polyembryony that contains a *MiRWP/MiRKD4* gene encoding an RWP-RK domain transcription factor. This gene is already recognized for its potential role in polyembryony in *Citrus* (Ali et al. 2025). Therefore, the overlapping genetic mechanisms that induce multiple embryo formation in mango and *Citrus* are believed to represent a case of convergent evolution (Yadav et al. 2023).

#### Mangosteen, kokum (*Garcinia mangostana*, *G*. indica)

*G. mangostana* (mangosteen) and *G. indica* (kokum) are two tropical species. The first is known for its sweet, juicy fruit pulp, while the second is valued as an ingredient and condiment, especially for flavouring fish curry in coastal regions of India (Khanashyam and Gupta 2023). Both species show nucellar embryony despite having dioecious reproductive systems. The embryonic origin from sporophytic tissue has been confirmed by anatomical (Horn 1940) and histological studies of the seeds (Dike et al. 2020). In the study by Dike et al. (2020), bagged flowers did not set fruit without pollination, which confirms that pollen stimulation is necessary. Molecular marker analysis also supported this, as most F1 progenies displayed banding patterns identical to those of the maternal plant, indicating apomictic behavior (Dike et al. 2020).

#### Papaya (*Carica papaya*)

Although apomixis has not yet been confirmed in papaya, two studies suggest that some lines exhibit polyembryony. An early study of gynoecious and andromonoecious papaya lines observed seeds lacking endosperm, but containing one to four embryos near the micropylar end. These embryos were successfully rescued and germinated in vitro, and the resulting plants were identical to the mother plant, indicating apomixis (Vegas et al. 2003). In a recent study, analysis of a Costa Rican line revealed the development of embryo-like structures in bagged flowers without pollination, accompanied by transcriptional downregulation of *AGO9*, *MEA*, *RBR1*, and *SWA1*, which regulate DNA methylation, chromatin remodeling, and rRNA processing (Acevedo-Benavides and Bolaños-Villegas 2021). These observations, although not definitive, suggest that papaya may harbor genotypes with partial or latent polyembryony capacity.

#### Pepper (*Capsicum annuum*)

In a study by Nowaczyk (1987), spontaneous polyembryony in pepper cultivars and their F₁ and F₂ hybrids revealed twin plants in the F₂ progeny, resulting from two-embryonic seeds. The main source of additional embryos was splitting polyembryony, in which diploid embryos of maternal origin developed alongside sexual embryos in twin pairs. However, it remains unclear whether these embryos arose through adventitious embryony or from embryogenesis of embryo cells with reduced chromosome numbers, followed by spontaneous diploidization. No further supporting research on the occurrence of apomixis in pepper has been found in the literature.

#### Prickly pear (*Opuntia ficus-indica*)

Prickly pear cactus has been reported to show polyembryony (Kaaniche-Elloumi et al. 2013). In the study by Mondragon-Jacobo (2001), 17 breeding populations of Mexican origin all showed the presence of apomictic seedlings, especially after self-fertilization. It was reported that apomictic reproduction in prickly pear occurs alongside sexual seed formation, with polyembryony frequencies of 0.5–20% and a prevalence in polyploid accessions (Mondragon-Jacobo 2001; Espinosa Henríquez 2017).

#### Sichuan pepper (*Zanthoxylum bungeanum*, *Z*. *armatum)*

Besides the genus *Citrus, Z. bungeanum* and *Z. armatum in the* Rutaceae family, both known as Sichuan pepper, can produce viable seeds through apomixis without needing fertilization (Fei et al. 2021a, b; Wang et al. 2021; Ang 2025). Recent cytological observations suggest sporophytic apomixis. Specifically, some embryos originate from nucellar cells, forming indefinite embryonic primordia that later develop into adventitious embryos (Fei et al. 2021b). Additionally, a combination of techniques, including RNA sequencing and analysis of microRNA interactions with their target genes, was used to explore the dynamic regulatory mechanisms of apomixis in *Z. bungeanum* (Fei et al. 2021a).

### Pharmaceutical

#### Guggul (*Commiphora wightii*)

The medicinal and aromatic fruit tree native to India, *C. wightii*, is valued for its oleo-gum-resin (Kavane et al. 2021). Sporophytic apomixis has been reported in this species for the first time by Gupta et al. (1996) and Gupta et al. (1998) in approximately 50% of the analyzed seeds, along with the occurrence of autonomous endosperm formation, which is considered very rare. Later studies further examined evidence of sporophytic apomixis using controlled pollination, histology, flow cytometry, and fluorescence-activated cell sorting (FACS) analyses (Geetha et al. 2013; Kavane et al. 2021, 2022). These analyses clarified that endosperm formation in *C. wightii* can occur either through fertilization or autonomously, confirming the facultative apomictic nature of the species, with fruit set ranging from 1.1% to 20% in 43 accessions (Kavane et al. 2022).

### Gametophytic apomixis in crops: natural apomicts and mutants with elements of apomixis

For functional gametophytic apomixis, apomeiosis, parthenogenesis, and autonomous endosperm formation must all be coordinated within the same reproductive pathway (Asker 1980; Grimanelli et al. 2001; Bicknell and Catanach 2015; Barcaccia et al. 2020; Wang and Underwood 2023; Hörandl 2024). Many natural apomicts are described as facultative when they exhibit the first two components of apomixis: apomeiosis and parthenogenesis, but still require fertilization of the central cell for endosperm development (pseudogamy). In contrast, obligate apomicts form the endosperm autonomously, without fertilization, meaning that all three components of apomixis: apomeiosis, parthenogenesis, and autonomous endosperm development, are coupled (Koltunow and Grossniklaus 2003; Pupilli and Barcaccia 2012; Hojsgaard and Hörandl 2015).

*Apomeiosis* is the initial key component of gametophytic apomixis, involving the avoidance of meiosis, which results in an unreduced (2n) female gametophyte (Hörandl 2024). Two main subtypes are recognized: diplospory, where the megaspore mother cell directly forms an unreduced embryo sac without meiotic reduction (Cornaro et al. 2023), and apospory, in which the embryo sac develops from nucellar (2n) cells next to the megaspore mother cell (Conner et al. 2025).

*Parthenogenesis*, which is the development of an embryo from an unfertilized egg, is a key step in apomictic reproduction. From the earliest insights, parthenogenesis has been shown to occur in species, such as *Taraxacum* and *Hieracium* (Murbeck 1904; Bierzychudek 1985). Research on natural apomicts indicates that parthenogenesis often co-occurs with apomeiosis, but can also happen independently and separately from autonomous endosperm formation. Evidence for this comes from multiple apomictic species, including *Taraxacum officinale* (Van Dijk et al. 1999), *Erigeron* annuus (Noyes 2000, 2005; Noyes and Rieseberg 2000), *Poa pratensis* (Albertini et al. 2001), *Hypericum perforatum* (Barcaccia et al. 2006), *Megathyrsus maximus* (Kaushal et al. 2008), and *Hieracium* spp. (Catanach et al. 2006; Ogawa et al. 2013). For an extensive discussion of parthenogenesis, see Vijverberg et al. (2019). However, a major turning point in apomixis research was the identification of the *BBM1* and *PAR* genes, which are responsible for parthenogenesis in *Cenchrus squamulatum* and *Taraxacum officinale*, respectively, and have been efficiently incorporated into synthetic apomixis studies (Conner et al. 2015; Khanday and Sundaresan 2021; Underwood and Mercier 2022; Huang et al. 2024; Song et al. 2024).

*Autonomous endosperm formation* is the third and rarest element of gametophytic apomixis, where the central cell develops into nutritive tissue without fertilization (Hands et al. 2016; Rojek and Ohad 2023). Most apomicts, such as many *Paspalum* and *Urochloa* accessions, require pollination for central-cell fertilization (pseudogamy), even if the embryo develops parthenogenetically (Valle and Savidan 1996; Naumova et al. 1999; Zorzatto et al. 2010; Ortiz et al. 2013, 2020; Delgado et al. 2014; Depetris et al. 2018). True autonomous endosperm formation is reported mainly in certain *Hieracium* and *Taraxacum* species, but not only, allowing full seed development without pollen (Cooper and Brink 1949; van Baarlen et al. 2002; Rodrigues et al. 2008; Ogawa et al. 2013; Henderson et al. 2017; Van Dijk et al. 2020). For an up-to-date and comprehensive overview of all genes involved in autonomous endosperm development, see Rojek and Ohad (2023).

In the following sections, natural apomictic crops exhibiting gametophytic apomixis (in its apospory or diplospory forms) and crop mutants with elements of apomixis (apomeiosis, parthenogenesis, autonomous endosperm development), are summarized in Table 2.

**Table 2:** List of crops with natural occurrence of gametophytic apomixis and mutants with elements of gametophytic apomixis

Common name	Species name	Plant Family	General Features	Apomixis Element/s	Ploidy	Apomixis Occurrence	Methodologies	References
*Agrifood*								
Banana	*Musa acuminata*	Musaceae	Monocot, Herbaceous, Monoecious, Perennial	Apomeiosis	Polyploid	NR	Prevention from pollination, flow cytometry, phylogenomic analyses	(Okoro et al. 2011; Lin et al. 2024)
Brambles*	*Rubus* ser. *Glandulosi*	Rosaceae	Eudicot, Shrub, Hermaphrodite, Perennial	Apospory, Diplospory	Polyploid	Genotype-dependent, reaching > 95% of apomixis	Molecular marker, flow cytometry analyses	(Šarhanová et al. 2012; Krahulcova et al. 2013; Sochor et al. 2015, 2024a, b)
Cassava	*Manihot esculenta*	Euforbiacee	Eudicot, Shrub, Monoecious, Perennial	Apospory	Diploid	2% of aposporous sacs	Molecular marker, histological, segregation analyses	(Nassar 1994; Nassar et al. 1998a, b)
Chinese chive	*Allium tuberosum, A. ramosum*	Amaryllidaceae	Monocot, Herbaceous, Hermaphrodite, Perennial	Diplospory	Polyploid	7–98% of diplospory	Histological, segregation analyses	(Kojima and Nagato 1992, 1997; Kojima et al. 1994; Yamashita et al. 2012)
Crabapples	*Malus* spp.	Rosaceae	Eudicot, Tree, Hermaphrodite, Perennial	Apospory	Di-triploid	78.7% of apomictic fruit set	Histological analysis, flow cytometry, expression analyses	(Sax 1959; Qu et al. 2006; Feng et al. 2023; Liu et al. 2023)
Date plum	*Diospyros lotus*	Ebenacee	Eudicot, Tree, Dioecious, Perennial	NR	Diploid	55% of apomictic seed set	White-latex pollen isolation technique, pollen tube germination tests, molecular markers	(Ikeda and Sugiura 2000; Zhou et al. 2022)
Maize	*Zea mays*	Poacee	Monocot, Cereal grass, Monoecious, Annual	Apomeiosis, Parthenogenesis, Autonomous Endosperm	Diploid	> 50% of apomixis rate	Prevention from pollination, histological, chromosome counting, half-tetrad, segregation analyses, progeny tests	(Tyrnov and Enaleeva 1983; Leblanc et al. 1995; Enaleeva et al. 1997; Grimanelli et al. 1998; Volokhina et al. 2021; Moiseeva et al. 2024; Chumak et al. 2025)
Pearl millet	*Cenchrus americanus*	Poaceae	Monocot, Cereal grass, Hermaphrodite, Annual	Apospory	Polyploid	6.4–13.2% of seed set in the genotype 169–46	Molecular markers, histological, flow cytometry analyses, FISH, GISH, segregation analysis, progeny tests	(Ozias-Akins et al. 1993, 1998, 2003; Morgan et al. 1998; Roche et al. 2001; Singh et al. 2010)
Potato	*Solanum tuberosum*	Solanaceae	Dicot, Herbaceous, Hermaphrodite, Perennial	Apomeiosis	Polyploid	NR	Histological, chromosome counting, segregation analyses, progeny tests	(Mok and Peloquin 1975; Douches and Quiros 1988; Peloquin et al. 1989; Werner and Peloquin 1990)
Rice	*Oryza sativa*	Poaceae	Monocot, Cereal grass, Hermaphrodite, Annual	Apomeiosis	Diploid	NR	Molecular marker, histological, flow cytometry analyses	(Li et al. 1964; Jena and Khush 1990; Khush et al. 1994; Hai et al. 2007; Makabe et al. 2022)
Rapeseed	*Brassica* spp.	Brassicaceae	Eudicot, Herbaceous, Hermaphrodite, Annual	Apomeiosis	Polyploid	0.04–5.21% of unreduced gametes	Segregation analysis, chromosome counting	(Addo Nyarko et al. 2025)
Sea buckthorn	*Hippophae rhamnoides*	Elaeagnaceae	Eudicot, Shrub/small tree, Dioecious, Perennial	Apospory, Adventitious embryony	Diploid	1.61–13.48% of apomictic seed set	Prevention from pollination, histological analysis	(Mangla et al. 2015; Ali and Kaul 2017)
Sorghum	*Sorghum bicolor*	Poacee	Monocot, Monoecious with perfect and/or unisexual flowers, annual	Apospory	Polyploid	3–14% of aposporous embryo sacs	Histological, flow cytometry analyses, progeny tests	(Hanna et al. 1970; Reddy et al. 1980; Tang et al. 1980; Rana et al. 1981; Carman et al. 2011; de Morais Cardoso et al. 2017; Nassary et al. 2025)
Strawberry	*Fragaria x ananassa*	Rosaceae	Eudicot, Herbaceous, Hermaphrodite, Perennial	Parthenogenesis	Polyploid	NR	Histological analysis	(Dziadczyk et al. 2011; Leszczuk et al. 2018; Zini 2025)
Strawberry guava	*Psidium cattleianum*	Myrtaceae	Eudicot, Tree, Dioecious/Polygamous flowers, Perennial	Diplospory	Polyploid	64.2% of pseudogamous apomixis	Histological, flow cytometry analyses	(Souza-Pérez and Speroni 2017; Da Luz-Graña et al. 2025)
Sugar beet	*Beta vulgaris*	Amaranthaceae	Eudicot, Herbaceous, Hermaphrodite, Biennial	Apomeiosis, Parthenogenesis	Polyploid	1% to 61% of apomictic seed set	Molecular markers, isozyme analysis, flow cytometry, comparative proteomics	(Jassem 1976; De Bock 1985; Zhu et al. 2009; Szkutnik 2010)
Sunflower	*Helianthus annuus*	Asteraceae	Eudicot, Herbaceous, Hermaphrodite, Annual	Apospory	Polyploid	61.9% expressivity of apospory in triploids	Histological, flow cytometry, segregation analyses, SNP-based progeny tests	(Voronova 2014; Menendez et al. 2022; Lv et al. 2025; Pessino et al. 2025)
Tea tree	*Camellia sinensis*	Theaceae	Eudicot, Shrub, Hermaphrodite, Perennial	Apomeiosis, Parthenogenesis	Diploid	NR	Interspecific crosses, seed count analysis	(Takeda 1990; Hembree et al. 2019)
Walnut	*Juglans regia*	Juglandaceae	Eudicot, Deciduous tree, monoecious, dichogamous, perennial	Apospory	Diploid	2.77–44.89% of apomixis rate	Prevention from pollination, histological, transcriptomic, metabolomic analyses	(Wu et al. 2010; Zhang Qiang et al. 2014; Bao et al. 2025)
*Pharmaceutical*								
St. John’s wort*	*Hypericum perforatum*	Hypericaceae	Eudicot, Shrub, Hermaphrodite, Perennial	Apospory	Polyploid	Genotype-dependent, reaching > 95% of apomixis	Molecular markers, histological, flow cytometry, segregation analyses	(Barcaccia et al. 2006; Schallau et al. 2010; Galla et al. 2011, 2013)
Castor bean	*Ricinus communis*	Euphorbiaceae	Eudicot, Shrub, Monoecious unisexual flowers, Perennial	NR	Diploid	20.09% to 92.31% of apomictic seed set	Prevention from pollination, expression analysis	(Setayeshnasab et al. 2024, 2025)
*Animal feed/Forage crops*								
Alfalfa	*Medicago sativa*	Fabaceae	Eudicot, Herbaceous, Hermaphrodite, Perennial	Apomeiosis	Polyploid	NR	Histological, half-tetrad, segregation analyses	(Barcaccia et al. 1997b, c, 2000a, 2001, 2003; Citterio et al. 2005; Palumbo et al. 2024)
Bahiagrass*, Crowngrass*	*Paspalum notatum, P. simplex*	Poaceae	Monocot, Grass, Hermaphrodite, Perennial	Apospory, Unbalanced endosperm development	Polyploid	Genotype-dependent, reaching > 95% of apomixis	Molecular markers, histological, flow cytometry, segregation analyses	(Pupilli et al. 2001, 2004; Laspina et al. 2008; Ortiz et al. 2017, 2020; Depetris et al. 2018; Colono et al. 2022; Bellucci et al. 2023)
Buffelgrass*	*Cenchrus ciliaris*	Poaceae	Monocot, Grass, Hermaphrodite, Perennial	Apospory	Polyploid	Genotype-dependent, reaching > 95% of apomixis	Molecular markers, histological, flow cytometry, segregation analyses	(Hignight et al. 1991; Vielle-Calzada et al. 1996; Roche et al. 1999; Jessup et al. 2003; Ozias-Akins et al. 2003; Akiyama et al. 2005; Goel et al. 2006; Huo et al. 2009)
Guinea grass*	*Megathyrsus maximus*	Poaceae	Monocot, Grass, Hermaphrodite, Perennial	Apospory	Polyploid	Genotype-dependent, reaching > 95% of apomixis	Molecular markers, histological, flow cytometry, segregation analyses	(Savidan 1982; Kaushal et al. 2008; Yamada-Akiyama et al. 2009; Deo et al. 2020)
Kentucky bluegrass*	*Poa pratensis*	Poaceae	Monocot, Grass, Hermaphrodite, Perennial	Diplospory	Polyploid	Genotype-dependent, reaching > 95% of apomixis	Molecular markers, histological, flow cytometry, segregation analyses	(Naumova et al. 1993; Mazzucato et al. 1995, 1996; Barcaccia et al. 1997a; Albertini et al. 2001, 2004, 2005; Marconi et al. 2020)
Sheda grass*	*Dichanthium annulatum*	Poaceae	Monocot, Grass, Hermaphrodite, Perennial	Apospory	Polyploid	Genotype-dependent, reaching > 95% of apomixis	Molecular markers, histological, flow cytometry, segregation analyses	(Oke 1951; Celarier et al. 1958; Harlan et al. 1964; Reddy and d’Cruz 1969; De Wet and Harlan 1970; Bhat et al. 2010)
Signalgrass*	*Urochloa* spp.	Poaceae	Monocot, Grass, Hermaphrodite, Perennial	Apospory	Polyploid	Genotype-dependent, reaching > 95% of apomixis	Molecular markers, histological, flow cytometry, segregation analyses	(Miles et al. 1994; Valle and Savidan 1996; Naumova et al. 1999; Zorzatto et al. 2010; Ferreira et al. 2021; da Costa Lima Moraes et al. 2023)
Slimstem reedgrass*	*Calamagrostis* spp.	Poaceae	Monocot, Grass, Hermaphrodite, Perennial	Diplospory	Polyploid	Genotype-dependent, reaching > 95% of apomixis	Molecular markers, histological, flow cytometry, segregation analyses	(Nygren 1946, 1954; Greene 1984)
Weeping lovegrass*	*Eragrostis curvula*	Poaceae	Monocot, Grass, Hermaphrodite, Perennial	Diplospory	Polyploid	Genotype-dependent, reaching > 95% of apomixis	Molecular markers, histological, flow cytometry, segregation analyses	(Selva et al. 2017; Zappacosta et al. 2019; Carballo et al. 2021b, 2024; Pasten et al. 2022; Gallardo et al. 2025)

*Natural apomicts

*NR* not reported

### Agrifood

#### Banana (*Musa acuminata*)

Banana cultivars, mainly derived from interspecific hybridizations between *M. acuminata* and *M. balbisiana*, often exhibit triploidy (2n = 3x = 33) (Cheesman 1947; Simmonds and Shepherd 1955; Lin et al. 2024). Okoro et al. (2011) provided the first evidence of apomixis in *M. acuminata* by documenting seed set in isolated, bagged inflorescences that were not pollinated. Lin et al. (2024) further showed, using flow cytometry and phylogenomic analyses, that triploid bananas can produce unreduced female gametes via apomeiosis, which then fuse with haploid male gametes from diploid *M. balbisiana* to yield tetraploid offspring. This mechanism, known as the triploid bridge, is recognized for facilitating polyploidization in *Musa* species (Lin et al. 2024).

#### Brambles (*Rubus series Glandulosi*)

Flow cytometry seed-screen analysis of bramble fruits has shown 100% sexual reproduction in diploids, obligate unreduced embryo sac development in triploids, and mixed reproductive modes in tetraploids (Šarhanová et al. 2012). Notably, *R. bifrons* can switch between sexual and apomictic reproduction depending on environmental conditions (Šarhanová et al. 2012). Molecular and karyological studies suggest that European polyploid *Rubus* species originated from a small group of ancestral diploids, with frequent hybridization events driving reticulate evolution and the formation of multiple apomictic taxa (Krahulcova et al. 2013; Sochor et al. 2015). Geographic parthenogenesis is observed in tetraploid *R.* ser. *Glandulosi*, with apomicts restricted to Northwestern Europe and sexuals occurring throughout the rest of Europe and West Asia (Sochor et al. 2024a, b).

#### Cassava (*Manihot esculenta*)

Aposporous apomixis in cassava was initially discovered in interspecific hybrids and later confirmed through genetic studies using RAPD (Random Amplified Polymorphic DNA) markers, along with embryological and cytogenetic analyses (Nassar 1994; Nassar et al. 1998a, b). The frequency of facultative apomixis was low, estimated at around 2%, and was observed across multiple genotypes, including one derived from a different interspecific cross. Notably, apomictic behavior was observed in an F1 hybrid, indicating that apomixis can be directly transferred from wild relatives to cultivated cassava (Nassar et al. 1998a). The same study provided embryological evidence supporting apospory, with nucellar cells developing into embryo sacs. These findings highlight the potential of using apomixis in cassava breeding to preserve hybrid vigor clonally.

#### Chinese chive (*Allium tuberosum*, *A*. *ramosum*)

Apomixis in *A. tuberosum* and *A. ramosum* occurs predominantly via diplospory, with parthenogenesis occurring independently of pollination (Kojima and Nagato 1992; Yamashita et al. 2012). In *A. tuberosum*, cultivars show high rates of parthenogenesis (62–94%), and autonomous embryogenesis has been observed in both unreduced egg cells and egg-like antipodal cells (Kojima and Nagato 1992; Kojima et al. 1994). Genetic studies in *A. ramosum* further show that crosses between amphimictic and apomictic diploids produce triploid offspring with strong apomictic expression, whereas diploid offspring remain sexual (Yamashita et al. 2012).

#### Crabapples (*Malus* spp)

Apomixis has been observed in multiple *Malus species* (Rosaceae), particularly in polyploid groups (Sax 1959; Qu et al. 2006). It has been reported in at least 10 *Malus* species, including *M. hupehensis*, *M. sikkimensis*, *M. rockii*, *M. sieboldii*, *M. platycarpa*, *M. sargentii*, *M. xiaojinensis*, *M. lancifolia*, and *M. corolla* (Liu et al. 2014, 2023). In these species, apospory is the most common mechanism, with embryo sacs developing directly from nucellar cells and embryos forming from unreduced egg cells (Liu et al. 2014). In tea crabapple (*M. hupehensis* var. *pingyiensis*), both apomeiosis and parthenogenesis have been documented, with their genetic regulation seemingly involving distinct loci for each process (Liu et al. 2014). Transcriptomic studies of apomictic and sexual *Malus* species revealed that plant hormone signal transduction is a key pathway influencing apomictic reproduction (Liu et al. 2023). A newly discovered species, *M. shizongensis*, closely related to *M. hupehensis*, has also been shown to reproduce apomictically. Controlled bagging experiments demonstrated a high fruit set of 78.7%, indicating autonomous reproduction (Feng et al. 2023). Additionally, cytological results from the same study suggest that male gametophyte failure, along with reduced ovule fertilization, underpins the apomictic reproductive strategy of the species.

#### Date plum (*Diospyros lotus*)

Deviation from sexuality was observed in* D. lotus*, an important persimmon rootstock in North China (Zhou et al. 2022). In that study, a new white-latex pollen isolation technique was validated as a reliable alternative to sulfuric acid paper bagging, effectively blocking pollen germination and enabling large-scale germplasm screening. Using this method, 12 of 22 tested date plum accessions produced viable seeds after pollen isolation, suggesting apomictic reproduction (Zhou et al. 2022). In another study, seedlings analyzed with SSR (Simple Sequence Repeats) and AFLP (Amplified Fragment Length Polymorphism) markers further suggested asexual reproduction, with some progeny clonally derived from the maternal plant and others formed through sexual reproduction (Ikeda and Sugiura 2000).

#### Maize (*Zea mays*)

Maize exemplifies the loss of apomixis in cultivated plants. Its wild ancestor, *Tripsacum dactyloides*, and *Tripsacum–maize* hybrids retain the ability to reproduce asexually through apomixis, whereas cultivated maize has lost this ability, likely due to human-driven breeding (Matsuoka 2005; Leblanc et al. 2009; Belova et al. 2010; Grimanelli 2012; Moiseeva et al. 2024). Mutants with element/s of apomixis in maize have been found in the *elongate* mutant, where abnormal meiosis leads to unreduced female gamete formation (Nel 1975). Further research showed that diploid embryo sacs in *elongate1* result from the absence of meiosis II, with meiosis I proceeds normally, allowing recombination and chromosomal assortment (Barrell and Grossniklaus 2005). Insights on efforts to transfer apomixis from *Tripsacum* into maize and identified by molecular markers linked with the diplosporous mode of reproduction, were obtained by Leblanc et al. (1995). The following work by Grimanelli et al. (1998), analysed various generations of maize–*Tripsacum* hybrids and backcross derivatives for the phenotypical expression of the apomictic mode of reproduction and for the transmission of the chromosomal segment carrying the apomictic gene(s) suggesting that the gene or genes controlling apomixis are not inherited through haploid gametes, rather than not expressed in diploids. Importantly, through extensive crossing and selection, the first parthenogenetic maize line (AT-1), which exhibited a high and heritable rate of maternal parthenogenesis in embryo sacs, was developed (Tyrnov and Enaleeva 1983; Enaleeva et al. 1997; Kolesova and Tyrnov 2011). Several lines derived from AT-1, including AT-3, ATT, and ATTM, showed parthenogenesis and autonomous endosperm development at frequencies ranging from 6% to over 50%. Building on this, multiple studies have sought to identify candidate genes associated with apomictic development (Danilevskaya et al. 2003; Hermon et al. 2007; Garcia-Aguilar et al. 2010; Makarevitch et al. 2013; Li et al. 2014; Volokhina et al. 2021; Moiseeva et al. 2024). For instance, parthenogenetic development has been observed in the AT-4 maize line, where atypical expression of *ZmFie1* and *ZmFie2*—genes that typically regulate endosperm initiation—occurred in the absence of fertilization, supporting spontaneous embryo and endosperm formation (Moiseeva et al. 2024). Overall, maize remains a primary focus of apomixis research, reflecting its global significance as a staple crop, and importantly, it supports our leading idea that screening for natural apomixis-related mutants in crop species is a promising strategy.

#### Pearl millet (*Cenchrus americanus*)

Pearl millet (formerly *Pennisetum glaucum*) is a cereal staple food for more than 90 million farmers, mainly cultivated in sub-Saharan Africa and South Asia (Salson et al. 2023). First attempts for the transmission of apomixis in pearl millet were conducted by Ozias-Akins et al. (1993), identifying markers that co-segregate with apomixis in crosses between apomictic *Pennisetum* and sexual pearl millet. Additionally, the impressive work was continued by Morgan et al. (1998) transferring aposporous apomixis from *Pennisetum squamulatum* into sexual pearl millet, successfully producing an obligate apomictic genotype (169–46), however, with low seed set (6.4–13.2%). Subsequent intensive genetic studies, initially based on molecular markers and later complemented by FISH (fluorescence in situ hybridization) and GISH (genomic in situ hybridization) analyses, led to the identification and characterization of the apospory-specific genomic region (ASGR) (Ozias-Akins et al. 1998, 2003; Roche et al. 2001; Singh et al. 2010, 2022).

#### Potato (*Solanum tuberosum*)

Potato is the world’s most important non-cereal crop. Yet, genetic improvement has traditionally been slow due to the high heterozygosity of autotetraploid cultivars and the complexity of its reproductive system (Hojsgaard et al. 2024). Early and intensive genetic studies by Mok and Peloquin (1975), Ramanna (1979), Douches and Quiros (1988), Peloquin et al. (1989), Werner and Peloquin (1990), Jongedijk et al. (1991) demonstrated that certain potato species can produce genetically unreduced, highlighting their significance in breeding programs. Furthermore, Peloquin et al. (2008) estimated the efficiency with which unreduced gametes transmit heterozygosity, showing that first-division restitution (FDR) 2*n* gametes from diploids transmit almost 50% more heterozygosity than *n* gametes from the tetraploid potato. These findings indicate that the use of unreduced gametes in polyploids can be exploited for breeding purposes and for predicting the genetic consequences of 2*n* gametes in the resulting progeny.

#### Rice (*Oryza sativa*)

Rice is a staple crop of global importance and plays a pivotal role in the economies of many developing Asian countries (Muthayya et al. 2014; Mohapatra and Sahu 2021). The occurrence of unreduced gametes in rice was observed in early cytological studies by Li et al. (1964). The authors reported the formation of triploid progeny (2n = 36) following backcrossing of the interspecific hybrid *O. sativa* × *O. officinalis* to *O. sativa*. Analyses of meiosis in the F₁ hybrid revealed that, in some cases, a lack of synchronization between nuclear division and cytokinesis occurred during microsporocyte division, leading to the production of diploid microspores, indicative of unreduced (2n) gamete formation. Further support for this phenomenon was discussed by Jena and Khush (1990), who noted that occasional unreduced gametes produced by interspecific hybrids can be fertilized by normal haploid gametes, resulting in the formation of allotriploid progeny, representing an important mechanism for polyploid formation and gene introgression between Oryza species (Khush 1987, 1997; Khush et al. 1994).

On the other hand, Hai et al. (2007) described a rice line (SARII-628) producing twin seedlings, in which diploid–triploid twin plants occasionally emerged from the same grain. Among approximately 4,500 seedling pairs analyzed, several cases of diploid–triploid twins were identified. Genetic analyses indicated that the triploid individuals originated from natural homologous triploidization without detectable changes in DNA sequence. However, epigenetic modifications, such as altered DNA methylation patterns, were observed by MSAP (Methylation-Sensitive Amplification Polymorphism) analysis.

In wild rice, Makabe et al. (2022) shed light on the possible origin of a naturally occurring triploid BKK line, suggesting that it derived from hybridization between an unreduced (2n) female gamete from an interspecific hybrid (*O. longistaminata* × *O. rufipogon*) and a reduced (n) male gamete from *O. officinalis*.

#### Rapeseed (*Brassica* spp)

In *Brassica*, recent studies have suggested apomixis-like mechanisms by documenting the production of unreduced gametes after interspecific hybridization (Addo Nyarko et al. 2025). In crosses between *B. juncea* (AABB) and *B. napus* (AACC), third-generation hybrids (AABC) exhibited highly variable chromosome numbers (2n = 48–74), most likely attributable to unreduced gamete formation. Cytogenetic analysis revealed that unreduced gametes occurred at higher frequencies (0.04–5.21%) in AABC hybrids than in the parental species, suggesting that unreduced gametogenesis may facilitate genome stabilization and fertility restoration in polyploid lineages (Addo Nyarko et al. 2025).

#### Sea buckthorn (*Hippophae rhamnoides*)

*H. rhamnoides* is valued for its nutritional and medicinal properties, with berries rich in vitamins, antioxidants, and fatty acids. It is widely used in food, health, and cosmetic products (Suryakumar and Gupta 2011; Ciesarová et al. 2020). The species appears capable of both apospory and adventive embryony, with approximately 54% of progeny formed through syngamy, around 30% via the aposporous mode, and approximately 16% through sporophytic apomixis (Mangla et al. 2015). Facultative apomixis in this species is further supported by a study showing autonomous seed set ranging from 1.61% to 13.48% in bagging experiments (Ali and Kaul 2017).

#### Sorghum (*Sorghum bicolor*)

Sorghum is a major cereal crop valued for its resilience to climate stresses and its versatility in food, feed, and industrial uses (de Morais Cardoso et al. 2017; Espitia-Hernández et al. 2022; Nassary et al. 2025). In *S. bicolor*, aposporous apomixis has been observed in some genotypes (Hanna et al. 1970; Reddy et al. 1980; Tang et al. 1980). Carman et al. (2011) analyzed 65 accessions and found ≥ 3% aposporous embryo sac formation in 11 of them. Of these, ten were diploid, while one tetraploid accession showed the highest aposporous embryo sac frequency of 14% (Carman et al. 2011).

#### Strawberry (*Fragaria* × *ananassa*)

The earliest reports of apomixis in *F.* × *ananassa* date back to the 1980s (Niemirowicz-Szczytt 1984). More recently, pseudogamous apomixis was also identified in the European wood strawberry, *F.* vesca (Baturin 2009). Embryological studies of *F.* × *ananassa* cultivars, including ‘Selva’, ‘Mount Everest’, and ‘Senga Sengana’, found multicellular archespores in nearly all ovules, occasionally producing more than one developing megasporocyte, indicating the potential for apospory or diplospory (Dziadczyk et al. 2011; Leszczuk et al. 2018). Notably, in the study by Leszczuk et al. (2018), some ovules showed simultaneous meiotic and apomictic development, with multiple embryo sacs forming concurrently. Additional nucellar cell differentiation suggested a possible aposporic origin of unreduced gametophytes and strongly supported the existence of facultative apomixis in *Fragaria* × *ananassa* (Leszczuk et al. 2018). The variability and coexistence of sexual and apomictic embryo sac development pathways were later reported and explained in *F.* × *ananassa* cv. ‘Camino Real’ (Zini 2025).

#### Strawberry guava (*Psidium cattleyanum*)

*P. cattleyanum* Sabine is a shrub or small tree native to Brazil and widely cultivated in tropical and subtropical regions for its edible fruits and essential oils, which are natural compounds with antimicrobial and antioxidant properties (Scur et al. 2016; Soliman et al. 2016). Anatomical analyses of *P. cattleyanum* flower sections from early developmental stages through fruit formation revealed that the embryo sac arises via a non-meiotic pathway, supporting a diplosporic origin and representing the first proposal of this apomixis in the Myrtaceae (Souza-Pérez and Speroni 2017). Recent investigations into seed ploidy further confirmed apomixis in *P. cattleyanum*: of 492 seeds analyzed, 64.2% were determined to result from pseudogamous apomixis, and the embryo-to-endosperm ploidy ratio closely matched the expected 2:5 balance (Da Luz-Graña et al. 2025).

#### Sugar beet (*Beta* spp)

In the genus *Beta*, apomixis was first documented in wild species, such as *B. trigyna* and *B. lomatogona* (Jassem 1976; De Bock 1985). Maletskii and Maletskaya (1996) showed that, under low-temperature conditions, up to 46.14% of male-sterile plants and 75.53% of fertile plants produced seeds when isolated. They concluded that environmental factors, especially low temperatures, promote embryogenesis in apomictic embryos. In another study, both diplospory and apospory were reported, and interspecific crosses between sexually reproducing diploids (*B. macrorhiza*, *B. lomatogona*) and the hexaploid *B. trigyna* produced F1 hybrids with various ploidy levels (2n = 36, 37, 45), including both sexual and apomictic forms (Jassem 1976). Seilova (1996) experimentally confirmed facultative apomixis in sugar beet by developing a method to detect apomictic genotypes within diploid cultivar populations and successfully establishing apomictic inbred lines. This approach has since been used in breeding programs to accelerate the development of sugar beet cultivars (Szkutnik 2010). More recently, Zhu et al. (2009) identified apomictic phenotypes in interspecific hybrids of *B. vulgaris* and *B. corolliflora*.

#### Sunflower (*Helianthus annuus*)

An apospory-like phenotype was first observed in cytoplasmic male-sterile (CMS) sunflower lines pollinated by wild *Helianthus* species (Voronova 2014). Later, Menendez et al. (2022) reported embryological evidence for apospory and integumentary embryony in *H. annuus* inbred lines. Recently, Pessino et al. (2025) advanced this work by identifying a diploid sunflower line (Rf975) that naturally produces additional gametophytes resembling aposporous embryo sacs. The study examined the nature (reduced vs. unreduced) and developmental potential of these apospory-like structures by forming triploid (3x) BIII hybrids (2n + n) derived from Rf975. Morphological analyses confirmed that all triploids exhibited apospory, with an average expressivity of 61.9%. However, abnormal pollen development and decreased seed viability were also observed. In the same year, another study by Lv et al. (2025) reported and genetically confirmed the serendipitous discovery of parthenogenetic haploid seed formation in sunflower without the need for pollination. Although this phenomenon occurred at low frequency, in combination with previously reported evidence, it holds considerable promise for future research and plant breeding applications.

#### Tea tree (*Camellia sinensis*)

Few studies on the genus *Camellia* have reported evidence of unreduced gamete formation or possible parthenogenesis, particularly in interspecific crosses (Takeda 1990; Hembree et al. 2019). It was presumed that hybrids obtained by crossing *C. sinensis* with *C. sasanqua, C. brevistyla,* and *C. oleifera* might develop through parthenogenesis of a reduced gamete (Takeda 1990). Similar cases have been observed in other *Camellia* species and in some cultivars of the interspecific hybrid *C.* × *vernalis,* possibly due to unreduced gametes, interploid hybridization, or nonrecurrent apomixis (Hembree et al. 2019).

#### Walnut (*Juglans regia*)

Walnut, a perennial deciduous tree in the family Juglandaceae, is one of the world’s four most economically important nut crops, valued for its high nutritional value and diverse industrial uses (Wu et al. 2010; Bao et al. 2025). Reproductive studies have shown that walnut can undergo apospory, with rates varying widely among varieties, from 2.77% to 44.89% (Zhang Qiang et al. 2014; Xiong et al. 2019). More recent research using transcriptomics and metabolomics to compare apomictic and sexual embryos has provided new insights into the molecular regulation of this process in walnut (Bao et al. 2025).

### Pharmaceutical

#### St. John’s wort (*Hypericum perforatum*)

*H. perforatum* is a well-known medicinal plant valued for its wide range of bioactive compounds and extensively studied for the antidepressant properties of hypericin (e.g., Barnes et al. 2001; Whiskey et al. 2001; Henderson et al. 2002; Zou et al. 2004; Silva et al. 2005). It has also emerged as a promising model for studying apomixis. Key discoveries include the identification of the HAPPY (*Hypericum* APOSPORY) locus and the involvement of epigenetic regulators, including *IDN2*- and *FDM*-like genes, that contribute to the differentiation between sexual and asexual reproductive pathways (Barcaccia et al. 2006; Schallau et al. 2010; Galla et al. 2011, 2013, 2015, 2017, 2019; Basso et al. 2019). Despite these advances, the genetic control of apomixis in *H. perforatum* remains unclear. However, most genomic and transcriptomic studies have focused on the biosynthesis of medicinal metabolites rather than on their reproductive biology.

#### Castor bean (*Ricinus communis*)

Castor bean, an important oilseed crop, exhibits facultative apomixis (Setayeshnasab et al. 2024, 2025). A first study by Setayeshnasab et al. (2024) evaluated 30 genotypes over two consecutive years and found that apomixis contributed to seed production, with the highest percentages reaching 92.31%. Follow-up research examined the expression of eight candidate apomixis-related genes during three stages of flower development (Setayeshnasab et al. 2025). These results revealed distinct gene expression patterns between apomictic and sexually reproducing plants, offering insights into the molecular mechanisms underlying apomixis in castor bean.

### Animal feed/Forage crops

#### Alfalfa (*Medicago sativa*)

Cytological analyses of a diploid *M. sativa* subsp. *falcata* mutant, PG-F9, revealed that this genotype produces 2n egg cells via meiotic restitution, either by skipping the second division or through direct apomeiosis. This process bypasses normal meiotic reduction entirely (Tavoletti 1994; Barcaccia et al. 1997c, 2000a, 2003). While these findings confirm that unreduced gamete production occurs during development, progeny tests using morphological markers and molecular techniques, such as RAPD (Random Amplified Polymorphic DNA) and RFLP (Restriction Fragment Length Polymorphism), suggest that parthenogenesis associated with these 2n eggs is rare (Barcaccia et al. 1997c, 2001; Palumbo et al. 2024). More broadly, restitutional apomeiosis and haploid parthenogenesis have been observed across the *M. sativa–coerulea–falcata* complex, although complete apomictic development through seed cloning has not been observed (Palumbo et al. 2024). Recent transcriptomic data support cytological findings of unreduced gamete formation in alfalfa, showing differential expression of genes related to plant reproduction and ploidy regulation. These include candidates involved in meiosis, cell cycle control, and epigenetic regulation of germline fate (Palumbo et al. 2024). The observed changes in gene expression in alfalfa suggest that unreduced gamete formation is driven by specific molecular networks that control meiotic restitution and gametophyte development. However, stable parthenogenetic seed development has not yet been proven.

#### Bahiagrass, Crowngrass (*Paspalum notatum*, *P*. simplex)

The genus *Paspalum*, which has been the focus of apomixis research for over 50 years (Ortiz et al. 2013, 2020), has provided key insights into the molecular mechanisms controlling apospory, with particular attention to *P. simplex* and *P. notatum* (Caceres et al. 1999; Pupilli et al. 2004; Laspina et al. 2008; Ortiz et al. 2020). In *P. simplex,* an apomixis-controlling locus (ACL), defined by a single dominant allele, was identified (Pupilli et al. 2001, 2004). Significant progress in cytological, genomic, and transcriptomic data was achieved in *Paspalum notatum* (Ortiz et al. 2017; Podio et al. 2021). Some of the genes that emerged as promising candidates involved in apomixis pathways include: *QGJ*, whose expression in the ovule nucellus is linked to the formation of aposporous embryo sacs (AES) (Mancini et al. 2018); *TGS1*, which inhibits AES formation (Siena et al. 2014; Colono et al. 2019, 2022); and *ORC3*, which controls the development of maternal excess (4 m:1p) endosperm in apomictic genotypes (Bellucci et al. 2023). Moreover, a recent investigation of the candidate gene *TGS1* in *Arabidopsis* provided compelling evidence that this gene plays a key role in regulating apomeiosis (Siena et al. 2023). Overall, research on apomixis in *Paspalum* seems promising and has been facilitated by the availability of transcriptomic and genomic data generated through innovative sequencing technologies.

#### Buffelgrass (*Cenchrus ciliaris*)

*C. ciliaris* (formerly *Pennisetum ciliare*, commonly known as buffelgrass) is a facultatively apomictic, polyploid grass that has become a leading model for studying aposporous apomixis in the tribe Paniceae (Conner et al. 2025). Embryological studies describe aposporous embryo sacs arising from nucellar cells and identify pseudogamy as the reproductive mechanism (Hignight et al. 1991; Kaushal et al. 2010; Kumar and Chandra 2010). Subsequent genetic mapping localized the apospory-specific genomic region (ASGR), a single hemizygous, non-recombining chromosomal block inherited as a dominant factor, which has become a key reference locus for studying apomixis in grasses (Roche et al. 1999; Ozias-Akins et al. 2003; Akiyama et al. 2005; Goel et al. 2006). Transcriptomic and genomic studies have further identified candidate genes and small RNAs associated with cell fate regulation and meiosis suppression (Dwivedi et al. 2007; Huo et al. 2009). Collectively, *C. ciliaris* remains a well-characterized apomictic system in Poaceae, providing critical insights into the molecular, structural, and evolutionary foundations of aposporous apomixis (Vielle-Calzada et al. 1996; Jessup et al. 2003).

#### Guinea grass (*Megathyrsus maximus*)

Guinea grass (formerly *Panicum maximum*) is primarily a tetraploid, facultative apomict, and has become a model for studying aposporous gametophytic apomixis. Early cytological and embryological research established the occurrence and variation of facultative apospory across many accessions (Savidan 1982), while subsequent microarray-based studies identified differentially expressed genes, implicating regulatory and epigenetic networks rather than a single structural gene in controlling apomixis (Yamada-Akiyama et al. 2009). High-resolution linkage mapping using dosage-sensitive SNPs localized a locus that controls apospory (apo-locus) and produced markers useful for marker-assisted selection (Kaushal et al. 2018; Deo et al. 2020).

#### Kentucky bluegrass (*Poa pratensis*)

*P. pratensis* L. serves as a model for studying diplosporous apomixis in grasses, including both sexual diploids and facultative or obligate apomictic polyploids. Early embryological studies (Naumova et al. 1993) showed that unreduced embryo sacs develop directly from megaspore mother cells, bypassing meiosis. This confirmed that diplosporous development and pseudogamous fertilization coexist with varying degrees of sexuality in natural populations (Mazzucato et al. 1995, 1996; Barcaccia et al. 1997a, 2000b). Subsequent molecular research has identified apomixis-linked markers and genes, including *PpSERK* (*SOMATIC EMBRYOGENESIS RECEPTOR-LIKE KINASE*) and *APOSTART*, which are expressed differently in apomictic versus sexual genotypes (Albertini et al. 2004, 2005; Matzk et al. 2005; Marconi et al. 2020).

#### Sheda grass (*Dichanthium annulatum*)

*D. annulatum*, commonly known as angleton grass, is a polyploid tropical grass found in India that reproduces apomictically via apospory (Harlan et al. 1964; Reddy and d’Cruz 1969; De Wet and Harlan 1970; Bhat et al. 2010). Within the genus, tetraploid species, such as *D. annulatum*, *D. aristatum*, *D. caricosum*, and *D. fecundum,* are classified as facultative or obligate apomicts, while some hexaploid genotypes of *D. papillosum* and *D. aristatum* are obligate apomicts (Oke 1951; Celarier et al. 1958; Bhat et al. 2010).

#### Signalgrass (*Urochloa* spp.)

*Urochloa* (formerly *Brachiaria*) species are important tropical forage grasses that exhibit aposporous gametophytic apomixis (Valle and Savidan 1996). Most *Urochloa* species are polyploid and facultatively apomictic (e.g., *U. brizantha*, *U. decumbens*, *U. humidicola*), whereas their diploid relatives reproduce sexually, providing a natural comparative system (Ferreira et al. 2021). Valle and Savidan (1996) presented the first evidence of aposporous embryo sacs arising from nucellar cells, confirming the coexistence of pseudogamous apomixis and sexuality in the genus. In *Brachiaria* grass, a SCAR (Sequence Characterized Amplified Region) marker has been developed to predict apomictic behavior with 90% accuracy in the hybrid population (Miles et al. 1994). Furthermore, pioneering work used bulked segregant analysis with RFLP and radioactive mRNA fingerprinting to identify differentially expressed genes in apomictic versus sexual genotypes (Leblanc et al. 1997; Pessino et al. 1997, 1998). Later on, Zorzatto et al. (2010) identified RAPD markers linked to the aposporous apomixis locus (Apo). Multiple genetic maps have been constructed, with the most recent being a high-density linkage map developed in the hexaploid tropical forage grass *U. humidicola* (Worthington et al. 2021; da Costa Lima Moraes et al. 2023).

#### Slimstem reedgrass (*Calamagrostis* spp.)

*Calamagrostis* spp. are facultatively apomictic grasses native to North America (Nygren 1946). It was first reported that apomictic genotypes of *Calamagrostis* are usually pollen-free, in contrast to the abundant pollen in amphimictic strains (Nygren 1954), further confirming apomixis in this genus, which is closely associated with polyploidy. Although less studied than classic model genera, such as *Poa* or *Cenchrus*, they represent an interesting model of diplosporous gametophytic apomixis of the *Taraxacum* type (Greene 1984).

#### Weeping lovegrass (*Eragrostis curvula*)

*E. curvula* has become a model for diplosporous gametophytic apomixis because of its unique ‘*Eragrostis*-type’ embryo sac and a diverse group of sexual and facultative/obligate apomictic (mostly polyploid) cytotypes (Carballo et al. 2021b; Pasten et al. 2022). Recent research has focused on RNA-seq/miRNA profiling and methylation analyses to identify potential pathways that influence apomixis and sexual reproduction (Selva et al. 2017; Carballo et al. 2021b, a, 2024). Advances in genetic mapping and genomics have progressed from several high-density linkage maps to a consensus linkage map for tetraploid accessions, leading to the discovery of an apomeiosis-linked locus (APO) (Zappacosta et al. 2019; Gallardo et al. 2025).

### Genomic and transcriptomic resources available for natural apomicts and mutants with elements of apomixis

Understanding the molecular pathways and genes involved in apomictic reproduction increasingly relies on generating and integrating genomic and transcriptomic data. High-throughput sequencing methods enable the identification of candidate genes, regulatory networks, and epigenetic modifications that control the switch from sexual to apomictic development. These datasets are crucial for unraveling the complex molecular mechanisms underlying apomixis and for guiding future functional research. Therefore, we organized the available genomic and transcriptomic data for species that, to varying extents, exhibit apomixis (natural apomicts) and for mutants with apomixis elements (with one or more apomixis elements), as previously described in this review. From this standpoint, we extend our investigation to include apomictic species that are not classified as crops, thereby enabling a more balanced comparison of data availability between naturally occurring apomicts and sexual mutant crops that exhibit element/s of apomixis. The metrics gathered on the availability of deposited sequences as an indicator of current knowledge for each species are represented in Table 3.

**Table 3:** Metrics availability of genomics and transcriptomics data for natural apomictic species and for mutants with elements of apomixis in the NCBI repository GenBank

Species	Common usage	NCBI taxid	n. DNA SRA	n. RNA SRA	n. NCBI genomes	RefSeq Genome	RefSeq or GenBank ID	Level assembly
*Allium ramosum*	Agrifood	105309	1	1	0	no	–	–
*Allium tuberosum*	Agrifood	4683	74	51	0	no	–	–
*Beta vulgaris*	Agrifood	161,934	3539	1640	327	yes	GCF_026745355.1	Chromosome
*Brassica*	Agrifood	3705	35,906	28,567	90	yes	–	–
*Brassica napus*	Agrifood	3708	12,558	16,126	32	yes	GCF_020379485.1	Chromosome
*Brassica oleracea*	Agrifood	3712	5957	3011	16	yes	GCF_000695525.1	Chromosome
*Brassica rapa*	Agrifood	3711	13,977	7324	22	yes	GCF_000309985.2	Chromosome
*Camellia sinensis*	Agrifood	4442	3842	5394	21	yes	GCF_004153795.1	Scaffold
*Capsicum annuum*	Agrifood	4072	10,696	3261	25	yes	GCF_002878395.1	Chromosome
*Carica papaya*	Agrifood	3649	547	440	26	yes	GCF_000150535.2	Scaffold
Citrus*	Agrifood	2706	6923	6196	94	yes	–	–
*Citrus sinensis*	Agrifood	2711	601	1927	22	yes	GCF_022201045.2	Complete Genome
*Citrus* x* clementina*	Agrifood	85,681	126	109	1	yes	GCF_000493195.1	Scaffold
*Diospyros lotus*	Agrifood	55,363	795	101	4	yes	GCF_014633365.1	Chromosome
*Fragaria* x* ananassa*	Agrifood	3747	1965	1508	14	no		Complete Genome
*Garcinia indica*	Agrifood	547,469	1	3	0	no	–	–
*Garcinia mangostana*	Agrifood	58,228	11	63	0	no	–	–
*Helianthus annuus*	Agrifood	4232	5554	2162	11	yes	GCF_002127325.2	Chromosome
*Hippophae rhamnoides*	Agrifood	193,516	82	180	2	no	GCA_051362535.1	Chromosome
*Malus*	Agrifood	3749	11,654	6783	180	yes	–	–
*Malus domestica*	Agrifood	3750	9211	5080	79	yes	GCF_042453785.1	Complete Genome
*Malus sylvestris*	Agrifood	3752	110	49	6	yes	GCF_916048215.2	Chromosome
Mangifera indica*	Agrifood	29,780	1288	767	5	yes	GCF_011075055.1	Chromosome
*Manihot esculenta*	Agrifood	3983	7300	4258	23	yes	GCF_001659605.2	Chromosome
*Musa acuminata*	Agrifood	4641	1525	1034	9	yes	GCF_036884655.1	Chromosome
*Opuntia ficus-indica*	Agrifood	371,859	2	9	0	no	–	–
*Oryza sativa*	Agrifood	4530	132,091	45,527	172	yes	GCF_034140825.1	Complete Genome
*Psidium cattleianum*	Agrifood	375,274	9	0	0	no	–	–
Rubus*	Agrifood	23,216	1415	543	14	no	–	–
*Rubus argutus*	Agrifood	59,490	10	62	1	no	GCA_040183295.1	Chromosome
*Rubus idaeus*	Agrifood	32,247	266	302	5	no	GCA_047496405.1	Scaffold
*Solanum tuberosum*	Agrifood	4113	18,945	8217	97	yes	GCF_000226075.1	Scaffold
*Sorghum bicolor*	Agrifood	4558	20,672	14,388	26	yes	GCF_000003195.3	Chromosome
*Syzygium paniculatum*	Agrifood	219,897	1	1	0	no	–	–
*Zanthoxylum armatum*	Agrifood	67,938	62	231	1	no	GCA_041053305.1	Chromosome
*Zanthoxylum bungeanum*	Agrifood	328,401	106	157	2	no	GCA_051990225.1	Scaffold
*Zea mays*	Agrifood	4577	109,057	55,448	187	yes	GCF_902167145.1	Chromosome
*Juglans regia*	Agrifood/Industrial	51,240	1538	900	8	yes	GCF_001411555.2	Chromosome
Calamagrostis neglecta*	Animal feed/Forage crops	395,286	278	0	0	no	–	–
Cenchrus americanus*	Animal feed/Forage crops	4543	4247	1072	14	no	GCA_963924085.1	Chromosome
Cenchrus ciliaris*	Animal feed/Forage crops	35,872	101	52	0	no	–	–
Dichanthium annulatum*	Animal feed/Forage crops	106,888	1	7	0	no	–	–
Eragrostis curvula*	Animal feed/Forage crops	38,414	80	18	1	no	GCA_007726485.1	Chromosome
*Medicago sativa*	Animal feed/Forage crops	3879	7351	3048	26	no	GCA_051527215.1	Complete Genome
Megathyrsus maximus*	Animal feed/Forage crops	59,788	275	61	0	no	–	–
Paspalum notatum*	Animal feed/Forage crops	147,272	55	69	2	no	GCA_022530915.1	Chromosome
Poa pratensis*	Animal feed/Forage crops	4545	23	310	1	no	GCA_947311845.1	Scaffold
*Urochloa* spp.*	Animal feed/Forage crops	37,562	241	192	3	no	–	–
*Commiphora wightii*	Industrial/Pharmaceutical	246,360	4	0	0	no	–	–
*Ricinus communis*	Industrial/Pharmaceutical	3988	1540	260	2	yes	GCF_019578655.1	Chromosome
Hypericum perforatum*	Pharmaceutical	65,561	56	261	4	no	GCA_965644135.1	Chromosome
Boechera holboellii*	none	93,890	8	5	0	no	–	–
Dryopteris affinis*	none	239,547	3	13	0	no	–	–
Erigeron annuus*	none	91,248	7	3	0	no	–	–
Hieracium spp*	none	102,745	127	0	0	no	–	–
Pilosella*	none	122,543	248	14	1	no	–	–
*Pilosella piloselloides*	none	1,628,030	214	12	1	no	GCA_040114295.1	Scaffold
Ranunculus auricomus*	none	568,533	490	0	0	no	–	–
Taraxacum officinale*	none	50,225	140	121	0	no	–	–

*Natural apomicts

We recorded the number of DNA and RNA accessions in the NCBI SRA (Sequence Read Archive) database, the number of genomes deposited in NCBI GenBank, and whether a genome is available in the secondary RefSeq database. When apomixis or apomictic element/s have been reported in multiple, unspecified species within the same genus, the table reports metrics at the genus level and for species with a genome available (in the case of *Rubus*, *Paspalum*, *Pilosella*, and *Urochloa*) or, when several genomes are available, for species represented in RefSeq (*Citrus*, *Brassica*, *Malus*).

At first glance, the number of genomes deposited for natural apomict species is very low, and no species currently has a genome listed in RefSeq, with the exception of two major cultivated crops, *Citrus* spp. and mango. This may indicate a delay in research on apomixis in utilizing genomic resources—resources that are, by necessity, essential for identifying genetic variants and transcriptional mechanisms involved in reproductive strategies. In this sense, the current lack of available resources may limit progress in apomixis research within natural apomicts.

In contrast, when examining resources for sexual species with the occurrence of apomictic elements, we find significantly higher numbers, mostly aligned with the economic importance of each crop. This extensive body of existing knowledge, developed and utilized for other purposes, provides a strong foundation for further research into apomictic mechanisms that have received limited attention thus far.

## Conclusions

**Fig. 3:**
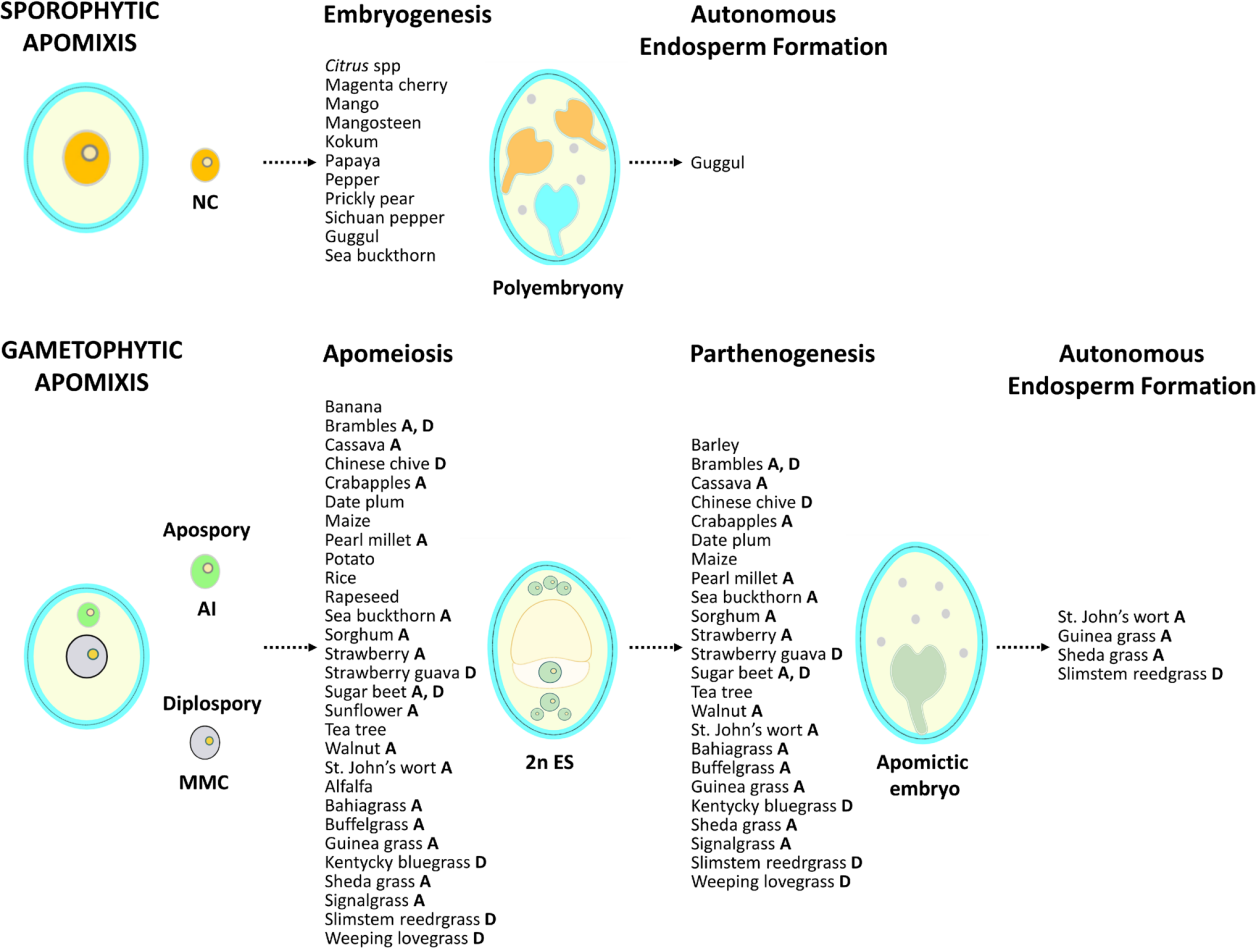
Simplified schematic representation of apomixis in crop plants, including both sporophytic and gametophytic types. In sporophytic apomixis, NC represents the nucellar cell. In contrast, in gametophytic apomixis, AI (Apospory Initial cell) indicates apospory, and Megaspore mother cell (MMC) indicates diplospory, while ES represents the embryo sac. Listed crops are annotated with an initial A and/or D when apospory or diplospory, respectively, has been reported. Cases of autonomous endosperm formation are included where documented, although pseudogamous development is likely the prevalent form. Castor bean (*R. communis*) is not included, as the cytological origin of its apomixis remains unclear

As we embark on this paradigm shift, it is essential to conduct a systematic review of the existing literature, encompassing both theoretical frameworks and empirical evidence pertinent to apomixis in major sexual crops. In this context, we highlight a considerable number of crop mutants that exhibit elements of apomixis (Fig. 3), thereby emphasizing the need to directly explore and investigate apomixis in this direction. This comprehensive process will significantly enhance our understanding of the genetic and environmental factors that influence the potential for apomixis.

Considering the constraints from decades of apomixis research, it is compelling to focus on identifying asexual reproductive mutants in sexual crops rather than only studying wild apomictic species and developing synthetic methods. Despite extensive efforts, with the exception of *BBM1,* findings from wild apomictic models to induce apomictic reproduction in agriculturally relevant crops have largely proved unsuccessful. Recent advances in genetic engineering, however, provide promising opportunities to create synthetic apomixis pathways in sexual crops. Notably, work manipulating the *MiMe* system and the *BBM1* gene in rice shows potential for developing asexual seed propagation mechanisms. Yet, while these methods offer a foothold in asexual reproduction, they do not fully capture the complex genetic frameworks controlling natural apomixis. This highlights the need for a paradigm shift toward understanding and engineering complex traits within the context of sexual crop species.

Moreover, the research landscape is rapidly evolving with the advent of modern genomic technologies that enable comprehensive screening of natural populations. Implementing these technologies in domesticated polyploid crop species could significantly enhance the identification of genetic variants that underlie critical apomictic traits. Such a transition would not only advance the ambitious goal of establishing apomictic mechanisms in commercially relevant crops, but also illuminate the pathways through which apomixis can enhance agricultural sustainability by preserving superior genotypes and stabilizing yields across generations and seasons.

In conclusion, the shift toward studying apomictic mutants in crops marks a significant shift in perspective that could transform apomixis research. Focusing on sexually reproducing species, especially polyploids, opens new opportunities for practical, innovative applications that address current agricultural challenges. This strategic change not only considers the complex realities of crop biology, but also aligns with broader goals of improving food security and agricultural resilience amid the risks posed by a constantly changing environment.

## Data Availability

All data collected in this study are included in the manuscript.
